# Bone Mineral Metabolism and Subsequent Hospitalization With Poor Quality of Life in Dialysis Patients

**DOI:** 10.5812/numonthly.14944

**Published:** 2014-01-20

**Authors:** Zohreh Rostami, Mahboobeh Sadat Hosseini, Mahboob Lessan Pezeshki, Farrokh Heidari, Behzad Einollahi

**Affiliations:** 1 Nephrology and Urology Research Center, Baqiyatallah University of Medical Sciences, Tehran, IR Iran; 2 Department of Nephrology, Tehran University of Medical Sciences, Tehran, IR Iran; 3 School of Medicine, Tehran University of Medical Sciences, Tehran, IR Iran

**Keywords:** Bone Density, Quality of Life, Renal Dialysis, Calcium, Phosphate

## Abstract

**Background:**

Significant impairment in health-related quality of life (HRQOL) among dialysis patients could be partly explained by some co-morbid disorders, such as chronic kidney disease-mineral and bone disorder (CKD-MBD). Also disturbance in calcium and phosphorus metabolism would increase mortality and morbidity. Therefore, further efforts to treat these abnormalities may improve the survival.

**Objectives:**

We designed a large multicenter population-based study in Iran to describe and assess the relation between HRQOL, hospitalization, and bone metabolism markers.

**Patients and Methods:**

We enrolled a total of 5820 dialysis patients from 132 dialysis centers in different parts of the country whom were volunteers to cooperate between October 2010 and August 2011. The Iranian adapted version of the Kidney disease quality of life-short form (KDQOL-SF^TM^) version 1.3 questionnaire was used to assess the health related quality of life. The clinical and demographic characteristics were gathered from patients’ data files.

**Results:**

The mean (SD) age of patients was 54.88 (16.36) years, and the range was 2 to 99 years. Of all patients, 43.1% were female. The scores of kidney disease component summary (KDCS), physical component summary, mental component summary, and total quality of life were significantly higher in the lower quartile of corrected serum calcium and higher quartile of serum parathyroid hormone (PTH) levels (P < 0.05). In a regression analysis of multilevel data, while corrected serum calcium level was associated with total KDCS and short form health survey (SF-36) scores after adjusting for other variables, hospitalization was directly correlated with serum phosphorus level and had reverse correlation with dialysis duration and quality of life.

**Conclusions:**

In the current study, quality of life was correlated with serum calcium level, calcium-phosphate product, and serum PTH level, while hospitalization was correlated only with serum phosphorus level. However, quality of life was inversely correlated with hospitalization.

## 1. Background

Mineral metabolism and bone disorders are associated with poor outcomes in dialysis patients (1). In addition, poor quality of life in this population has been publicized by several studies, and the health-related quality of life (HRQOL) has been considered as an independent predictor of death in these patients (2-6). Based on some evidences, the significant impairment in HRQOL in dialysis patients could be partly explained by some co-morbid disorders, such as chronic kidney disease–mineral and bone disorder (CKD–MBD) (1, 7, 8), in which disturbance in calcium and phosphorus metabolism would increase mortality and morbidity, and so further efforts to treat these abnormalities may improve survival (9).

During the last two decades, incredible attempts have been made to improve the mineral and bone metabolism abnormalities in dialysis patients (10). However, clinical trials were not adequate to show healthy end points such as hospitalization (11). Regarding new therapeutic agents, which are currently used in the management of mineral and bone disorder, the bone and mineral metabolism handling in patients with end-stage renal disease (ESRD) is still far from national kidney foundation dialysis outcomes quality initiative (K/DOQI) guidelines (11). Furthermore, new medications have had no significant survival benefits in quality of life defects (10). Therefore, achievement to a sustain level of serum calcium, phosphate, calcium-phosphate product, and parathyroid hormone (PTH) with current therapeutic strategies would be challenging (12). Thus, it is probable that disordered mineral metabolism could be associated with impaired HRQOL and must be modified (13) with new evidences.

The results of investigation on an adequate population of patients with ESRD are required to evaluate the effects of optimal serum PTH, calcium, and phosphorus levels control on clinical outcomes such as quality of life and hospital admission. So the findings of the current study may help to refine future recommendations for the treatment of long-term hemodialysis patients (9).

## 2. Objectives

To our knowledge, there was not a large multicenter population-based study carried out in Iran to describe and assess the relation between HRQOL, hospitalization, and bone metabolism markers within the gold standard values which were recommended by the K/DOQI guidelines among dialysis patients. Therefore, we designed a large multicenter study on Iranian dialysis patients for these proposes.

## 3. Patients and Methods

### 3.1. Study Design and Research Population

We performed a cross-sectional multicenter observational study of patients with ESRD in Iran. The protocol was approved in the ethical committee of our institution and a total of 5820 long-term hemodialysis (HD) patients were enrolled from 132 dialysis centers in different parts of the country whom were volunteer to cooperate between October 2010 and August 2011. Patients had to meet the following inclusion criteria: stable clinical condition, at least 3 months since the start of HD treatment, at least received HD 3 times per week with each session lasting 3 to 4 hours. We excluded patients that were hospitalized for an acute illness, had vascular access failure, or dialysis was performed via a temporary vascular access.

### 3.2. Data Collection

In this study, the data was collected on three major categories including demographic and clinical characteristics and HRQOL through reviewing the medical records and self-reported questionnaires. The considered demographic variables were age, gender, marital status, insurance status, employment status, and educational level. The educational level was ranked in four grades as uneducated, primary school level, high school level, and university level.

The gathered clinical character+boratory investigation results, dialysis duration (vintage), and hospitalization. Primary renal disease was classified into 7 subgroups as hypertension, diabetes mellitus, polycystic kidney disease, lupus nephritis, chronic pyelonephritis, others, and unknown. Blood laboratory measurements of interest included total serum calcium, phosphorus, PTH, alkaline phosphatase, hemoglobin, albumin, creatinine, and fasting blood sugar levels. All of these were measured using standard, provincially accredited laboratory techniques. Measured calcium was corrected for level of serum albumin with the following formula:

Corrected calcium = ([4albumin] × 0.8) + measured calcium

The Iranian adapted version of the kidney disease quality of life-short form (KDQOL-SF^TM^) version 1.3 questionnaire was used to assess health related quality of life. Details of translation and validation of KDQOL-SF^TM ^version 1.3 questionnaire have been described in other studies (3). The questionnaire is generally self-administered, so the patients could complete it by themselves during regularly scheduled dialysis sessions. Physicians or nurses were available for questions or brief explanation, if needed.

### 3.3. Instrument

Quality of life (QoL) was assessed by KDQOL-SF^TM ^1.3 questionnaire that included two separate questionnaires as short form health survey (SF-36), a generic measure, and kidney disease quality of life (KDQOL), a measure specific to patients with ESRD.

For SF-36, we calculated two summary scores from its eight scales. First, the physical component summary score (PCS), as the mean of physical functioning, role-physical, bodily pain, and general health perception scores. Second, the mental component summary score (MCS), as the mean of mental health, role-emotional, social functioning, and vitality scores.

KDQOL (ESRD-targeted Area) was used for calculating kidney disease component summary (KDCS) score. The KDCS was derived by 11 scales including symptoms and problems list, effects of kidney disease on daily life, burden of kidney disease, work status, cognitive function, quality of social interaction, sexual function, sleep, social support, dialysis staff encouragement, and patient satisfaction. Scores ranged from 0 to 100, the higher the score, the better the QoL.

### 3.4. Statistical Analysis

The demographic and clinical variables of the study population were expressed as frequency of each qualitative variable, and the mean SD) of the quantitative variables. Mineral metabolisms (serum measured calcium, corrected calcium, phosphorus, calcium-phosphate product, and PTH concentrations) were categorized by their quartiles and the following cut-off concentrations were considered: measured calcium, 8.4, 9, and 9.7 mg/dL; corrected calcium, 8.25, 8.95, and 9.5 mg/dL; phosphorus, 4.4, 5.4, and 6.5 mg/dL; PTH, 106, 267, and 525 pg/mL; and calcium-phosphate product, 39.50, 47.85, and 58.65. We examined the association between health quality of life score (PCS, MCS and KDCS) with mineral metabolisms by using one-way analysis of variance (ANOVA). Clinical and demographic variables that significantly associated with total HRQoL score were added to a univariate regression model. Then we constructed multivariable regression model and sequentially added significant associated variables to adjust for potential confounders on the relationship of mineral metabolisms with total HRQoL score. Regression model contained eight demographic and clinical variables as age, dialysis duration, gender, marital status, employment status, corrected serum calcium, albumin, and hemoglobin concentrations.

In second part, we classified hospitalization to five categories: 0, 1 to 3, 4 to 7, 8 to 14, and more than 30 days. Subsequently, we used one-way ANOVA to evaluate the correlation between hospitalization and mineral metabolisms, dialysis duration, and QoL. All statistical analyses were performed with SPSS version 18.0 and STATA version 12.0. P values of 0.05 or less were considered to indicate statistical significance.

## 4. Results

### 4.1. Baseline Characteristics

A total of 5820 patients were studied. The mean (SD) age of patients was 54.88 (16.36) years, and the range was 2 to 99 years. Of all patients, 43.1% were female, 52.7% were educated, and 71.9% were married. The primary kidney disease etiologies were hypertension (31.2%), diabetes mellitus (25.4%), chronic pyelonephritis (5.5%), polycystic kidney disease (4.7%), and lupus nephritis (2.6%). The mean (SD) dialysis vintage of patients was 38.79 (40.08) months (range 0.5 - 408 months). The complete demographic and laboratory data are shown in Table 1. 

**Table 1.** Baseline Characteristics

Variables	
**Age, mean ± SD, y**	54.8894 ± 16.36
**Dialysis duration, mean ± SD, d**	38.7991 ± 40.08
**Primary kidney disease, No. (%)**	
Hypertension	1746 (31.2)
Diabetes mellitus	1423 (25.4)
Polycystic kidney disease	263 (4.7)
Lupus nephritis	144 (2.6)
Chronic pyelonephritis	309 (5.5)
Others	694 (12.4)
**Educational level, No. (%)**	
Uneducated	2175 (47.3)
Primary school	1215 (26.4)
High school	1053 (22.9)
University	158 (3.4)
**Gender, No. (%)**	
Male	2175 (47.3)
Female	1215 (26.4)
**Marital status, No. (%)**	
Married	4183 (71.9)
Single	1517 (26.1)
**Employment status, No. (%)**	
Employed	664 (88.3)
Unemployed	5016 (11.7)
**Insurance status, No. (%)**	
No insurance	192 (3.4)
Public insurance	3580 (62.9)
Supplementary insurance	1920 (33.7)
**Creatinine, mean ± SD, mg/dL**	8.9971 ± 3.49
**Fasting blood sugar, mean ± SD, mg/dL**	133.2110 ± 76.33
**Albumin, mean ± SD, g/dL**	4.2602 ± 0.82
**Hemoglobin, mean ± SD, g/dL**	10.1638 ± 1.83
**Measured calcium, mean ± SD, mg/dL**	9.0021 ± 1.11
**Corrected calcium, mean ± SD, mg/dL**	8.8742 ± 1.15
**Phosphorus, mean ± SD, mg/dL**	5.6063 ± 1.63
**Parathyroid hormone, mean ± SD, pg/dL**	417.2948 ± 4716
**Alkaline phosphatase, mean ± SD, IU/L**	382.3884 ± 340
**Calcium ± phosphorus product, mean ± SD, mg^2^/dL^2^**	50.3173 ± 16.03

### 4.2. Quality of Life

Table 2 shows the scores for quality of life scales (PCS, MCS, and KDCS) by categories of serum calcium, corrected serum calcium, serum phosphorus, calcium-phosphate product and PTH concentrations. The scores of KDCS, PCS, MCS, and total QoL were significantly higher in the lower quartile of corrected serum calcium and higher quartile of PTH concentrations (P < 0.05). No other significant associations were found between scores for quality of life scales and mineral metabolisms, except the scores for SF-36 (PCS and MCS) that were significantly different (P values of 0.004 and 0.008, respectively) in calcium-phosphate product categories.

**Table 2.** Correlation Between Bone Metabolism Markers and MCS, PCS, KDCS and SF-36 + KDCS

Variable	KDCS ^a^		PCS ^a^		MCS ^a^		SF-36 + KDCS	
	Mean ± SD	P Value	Mean ± SD	P Value	Mean ± SD	P Value	Mean ± SD	P Value
**Measured calcium, mg/dL**		0.23		0.46		0.32		0.23
Total	51.08 ± 13.26		40.97 ± 20.03		45.99 ± 19.59		51.08 ± 13.26	
≤ 8.4	51.16 ± 12.99		41.13 ± 19.160		45.69 ± 19.25		51.16 ± 12.99	
8.5 to 9	50.73 ± 13.05		40.56 ± 20.12		45.44 ± 19.29		50.73 ± 13.05	
9.1 to 9.7	51.69 ± 13.32		41.64 ± 20.15		46.77 ± 19.77		51.69 ± 13.32	
> 9.7	50.83 ± 13.65		40.62 ± 20.72		46.15 ± 20.04		50.83 ± 13.65	
**Corrected calcium, mg/dL**		< 0.001		0.001		0.032		< 0.001
Total	58.71 ± 11.25		42.14 ± 20.62		47.42 ± 19.95		52.12 ± 13.18	
≤ 8.25	60.53 ± 11.79		44.54 ± 20.71		49.15 ± 20.23		54.03 ± 13.61	
8.26 to 8.95	58.09 ± 10.87		41.74 ± 20.01		46.82 ± 20.39		51.61 ± 12.86	
8.96 to 9.5	58.32 ± 10.83)		41.61 ± 20.65		47.01 ± 19.12		51.69 ± 12.73	
> 9.5	57.88 ± 11.29		40.64 ± 20.95		46.69 ± 19.98		51.12 ± 13.37	
**Phosphorus, mg/dL**		0.69		0.44		0.26		0.69
Total	51.05 ± 13.25		40.97 ± 20.06		45.93 ± 19.54		51.05 ± 13.25	
≤ 4.4	50.81 ± 13.52		40.98 ± 20.48		45.18 ± 19.39		50.81 ± 13.52	
4.5 to 5.4	50.94 ± 13.62		41.35 ± 20.89		46.04 ± 19.95)		50.94 ± 13.62	
5.5 to 6.5	51.37 ± 13.03		41.25 ± 19.48		46.63 ± 19.65		51.37 ± 13.03	
> 6.5	51.08 ± 12.80		40.20 ± 19.27		45.82 ± 19.11		51.08 ± 12.81	
**Ca × Pa product, mg/dL**		0.87				0.008		0.11
Total	57.85 ± 11.62		40.99 ± 20.05	0.004	45.95 ± 19.53		51.07 ± 13.25	
≤ 39.50	57.75 ± 11.93		40.36 ± 20.11		44.53 ± 19.11		50.53 ± 13.43	
39.51 to 47.85	57.70 ± 11.47		41.95 ± 20.53		46.53 ± 19.73		51.36 ± 13.37	
47.86 to 58.65	58.01 ± 11.57		41.95 ± 20.42		46.92 ± 19.98		51.56 ± 13.41	
> 58.65	57.96 ± 11.54		39.69 ± 19.02		45.84 ± 19.22		50.81 ± 12.79	
**PTH^a^**		0.050		0.008		0.002		0.007
Total	51.42 ± 14.00		39.81 ± 19.81		47.43 ± 21.38		51.42 ± 14.00	
≤ 106	56.77 ± 12.17		35.31 ± 18.88		42.77 ± 20.62		48.48 ± 13.89	
107 to 267	57.92 ± 11.51		40.57 ± 19.86		46.52 ± 21.75		51.33 ± 14.21	
267 to 525	58.86 ± 12.34		41.61 ± 19.61		49.23 ± 21.38		52.33 ± 13.99	

^a^ Abbreviations: Ca × P, calcium phosphate product; KDCS, kidney disease component summary; MCS, mental component summary; PCS, physical component summary; PTH, parathyroid hormone.

One level regression analysis showed that total scores of KDCS and SF-36 had significant association with age, dialysis vintage, gender, marital status, employment status, serum albumin, hemoglobin, corrected calcium, and parathyroid hormone concentrations. In multilevel regression analysis, corrected serum calcium was associated with total KDCS and SF-36 scores after adjusting for other variables, as is shown in Table 3. PTH was not in final regression model analysis because it had significant correlation (P value < 0.001) with corrected calcium (spearman’s correlation coefficient = -0.317)

**Table 3.** Multilevel Regression Analysis

Variable	One Level Regression Analysis			Multilevel Regression Analysis		
	B	95 % CI	P Value	B	95 % CI	P Value
**Age**	-0.19	-0.17 to -0.21	< 0.001	-0.15	-0.191 to -0.128	< 0 0.001
**Dialysis duration**	-0.02	-0.011 to -0.028	< 0.001	0.01	-0.020 to 0.004	0.198
**Gender**	-2.44	-3.132 to -1.756	< 0.001	-1.29	-2.442 to -0.145	0.027
**Marital status**	-4.13	-4.795 to -3.467	< 0.001	-0.51	-1.532 to 0.523	0.336
**Employment status**	-1.82	-2.150 to -1.496	< 0.001	-1.33	-1.868 to -0.809	< 0.001
**Corrected calcium**	-0.89	-1.297 to -0.492	< 0.001	-0.56	-0.985 to -0.137	0.010
**Albumin**	2.24	1.572 to 2.917	< 0.001	1.19	0.472 to 1.915	0.001
**Hemoglobin**	0.49	0.310 to 0.687	< 0.001	0.48	0.234 to 0.738	< 0.001

### 4.3. Hospitalization

**Figure 1.**
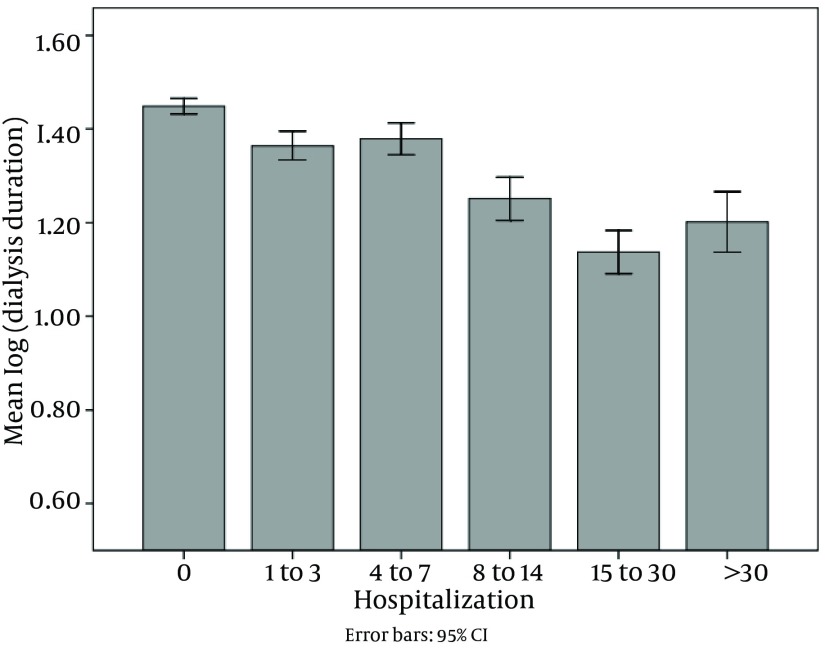
Correlation Between Hosspitalization and Dialysis Vintage (Dialysis Duration)

**Figure 2.**
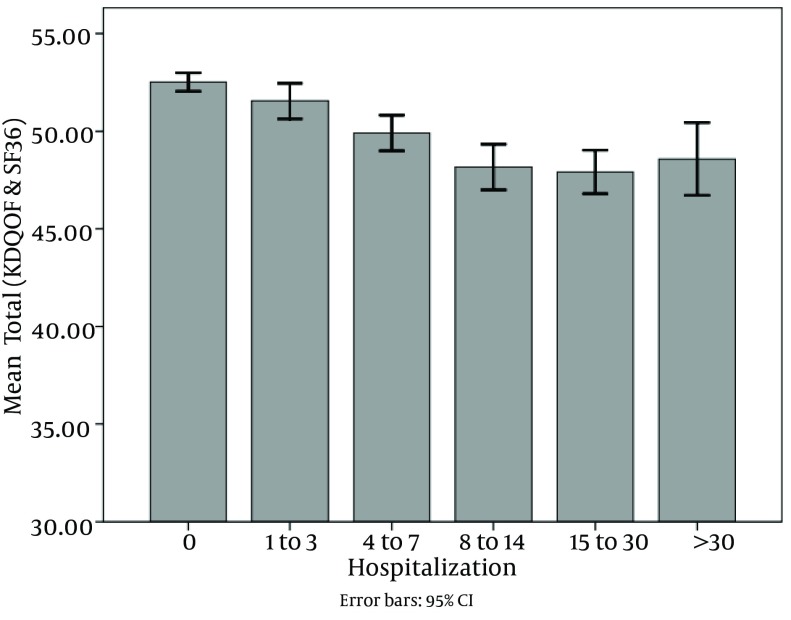
Correlation Between Hosspitalization and Kidney Disease Quality of Life Plus SF-36

Of all patients, 2801 ones were hospitalized (48.2%). The mean and mode of hospitalization period was 9.12 and 2 days, respectively, and the range was 1 to 105 days. Hospitalization had reverse correlation with dialysis vintage and quality of life. As shown in Figure 1 and Figure 2, dialysis duration and quality of life decreased in 5 categories of hospitalization.

**Figure 3.**
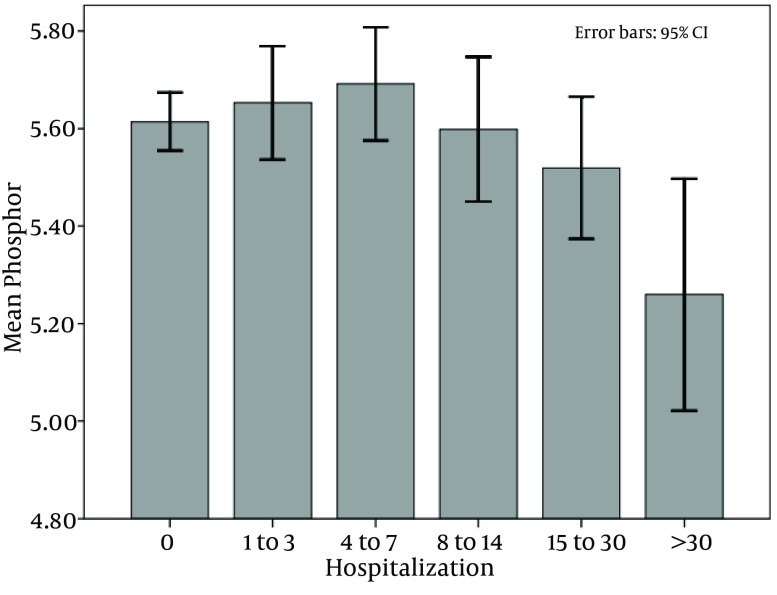
Corralation Between Hosspitalization and Phosphate Level

The mean of mineral metabolisms in these 5 categories were compared, and only the differences of serum phosphorus in the category of 4 to 7 days (mean = 5.69 ± 1.61), and 1 to 3 days (mean = 5.65 ± 1.62) with the category of more than 30 days (mean= 5.25 ± 5.2592) were significant (P values of 0.01 and 0.03, respectively) (Figure 3). 

## 5. Discussion

In the current study, quality of life had correlation with serum calcium, calcium-phosphate product, and PTH concentrations, while hospitalization was directly correlated with serum phosphorus level. However, quality of life had an inverse correlation with hospitalization. 

In an attempt to decrease the CKD-MBD related morbidity and mortality, clinical practice guidelines have been provided in some countries. Although, associations between mineral metabolic abnormalities including serum phosphorus, calcium, and PTH and mortality in dialysis patients (14) have been well documented, clinically relevant differences were observed among these guidelines, and safe amounts of serum calcium, phosphorus, and PTH yet need to be confirmed.

### 5.1. Health Related Quality of Life

We found that scores of KDCS, PCS, MCS, and total QoL were significantly higher in the lower quartile of corrected serum calcium and higher quartile of serum PTH concentrations. Abnormal mineral and bone metabolism is a common problem among chronic kidney disease (CKD) and is an important cause of morbidity, mortality, and reduced quality of life (15).

A previous study after adjusting for covariates demonstrated that both low and high serum phosphorus concentrations were associated with low PCS scores (13), because low serum phosphorus level as well as high level, may indicate malnutrition and anorexia due to poor dialysis adequacy which can cause wasting with inappropriate physical functioning (13). However, similar to our study, serum phosphorus level did not have any effect on MCS scores (13), and kidney disease symptoms. Nevertheless, another prior study demonstrated that high serum phosphorus levels management was associated with a considerable pill burden that was accompanying with lower quality of life in individuals with CKD (16).

In contrast to the mntioned study, our investigation revealed that better quality of life was associated with lower serum calcium level. Some studies have shown that high calcium levels were also associated with greater all-cause and cardiovascular mortality risk, and reduced mental health (17-19). Moreover, some studies have shown increased mortality rates in patients with low serum calcium levels, (17, 20, 21), while others failed to do so (14, 22).

We indicated that all aspects of QoL were associated with increased serum PTH level. However low serum PTH level was associated with worse PCS scores based on the findings of the mentioned study, it was not associated with kidney disease-related symptoms or with MCS scores (13).

The results of our study showed that a high serum PTH level induced by low serum calcium level in ESRD patients could lead to wellbeing and better quality of life. It could be explained with this fact that lower serum PTH levels might facilitate bone fracture and subsequently could predispose these patients to earlier mortality (23) and impaired QoL. However, single serum calcium level measurement which was done in our study might mislead possible associations between health status and serum calcium level. On the other hand, in some cases serum albumin level has not been measured, so in some cases corrected calcium levels were missing.

### 5.2. Hospitalizations

Almost half of our patients were at risk for hospitalization and the most important factor for hospital stay was high serum phosphorus level. In our study, hospitalization was increased with high phosphorus levels but the hospital stay was increased when phosphorus level was less than 5.2 mg/dL too. Moreover, greater dialysis vintage was associated with lesser hospitalization rate.

Previous studies demonstrated that disorders of bone mineral metabolism were independently related to increased cardiovascular diseases-associated mortality and morbidity risk (16), bone fracture risk and occurrence (15), soft tissue calcifications (24), and growth retardation in children (15) among dialysis patients (14, 25). Likewise, admission in hospital would have been increased in association with hyperphosphatemia, high serum calcium level, and markedly increased serum PTH levels (13, 14, 25, 26) even in community-based sample of individuals without baseline CKD (26). On the other hand, another study found that the high mortality risk might be observed in low serum PTH levels (26). Moreover, our study revealed, after adjustment for contributing factors, that hospitalization was correlated only with excess serum phosphorus level while serum calcium and PTH concentrations had no significant impact. A prior study has also shown that the higher levels of serum phosphorus were associated with increased mortality and cardiovascular-related hospitalization (27), independently of serum calcium and PTH levels (28). Recent studies support the hypothesis that these associations are mediated by vascular calcification (27, 28) in advanced kidney disease and diabetic patients (2). Hyperphosphatemia, arteriosclerosis, and vascular calcification are basic features of fibroblast growth factor 23 gene 2 and klotho gene 3 knockout models (28). A previous study revealed that serum phosphorus levels greater than 6.0 mg/dL were associated with up to 55% greater hospitalization risk and up to 3-fold greater mortality risk (29). There are several possible mechanisms that may describe the association of serum phosphorus levels with high cardiovascular disease risk. First, high serum phosphorus levels inhibit 1,25-dihydroxyvitamin D, and low levels of 1,25-dihydroxyvitamin D are associated with reduced cardiac contractility and greater vascular calcification. Second, high serum phosphorus level induces mineral deposition in vascular smooth muscle cells due to increased osteopontin expression, which can be augmented in the presence of high calcium-phosphate product. Third, it may be associated with high serum PTH level too (26, 28). In addition, recent studies revealed an associations between higher serum phosphorus levels with increased risk of infections in dialysis patients (2), since factors regulating bone metabolism could influence immune system maturation (27, 30).

In contrast to a previous study (31), our study revealed that hospital stay period was shorter in longer dialysis vintage. By the way, previous studies found an inverse relationship between vintage and death risk among prevalent hemodialysis patients (32-35). However, since there has been a selection bias, the correlation between dialysis vintage and outcome of patients with ESRD has been difﬁcult to deﬁne. Conversely, some studies have provided several explanations for these issues. First, it is hypothesized that the strategy including higher dialysate calcium concentrations and more calcium salts in prescriptions may have had an impact on the vascular calcification observed in longer duration of dialysis (36). Second, so many dialysis patients who may not be proper candidates for transplantation do live in steady state (37). Third, patients who are well enough to survive many years on dialysis may have some undesirable factors that might directly influence their survivals, but cannot be accounted for statistical analysis (37).

### 5.3. Limitation

Although we believe that our study had a large sample size, serum PTH level was not measured in all individuals, as well as we did not measure serum intact PTH levels. Information regarding comorbidities might be incomplete, and we did not have any data about disorders of bone metabolism. As in any observational study, there could be confounding factors by unmeasured covariates such as serum vitamin D level, serum parathyroid hormone level, and serum alkaline phosphatase concentration.

In addition, causality could not be established because of the observational design of this study. The cross-sectional nature of the study also limited the conclusions that could be drawn regarding causality ascertainment of hospitalization that was limited to follow-up period (27).

Since serum concentrations of serum phosphorus and PTH levels may change over relatively short time periods, single measurements might not accurately reflect time-averaged concentrations over the period which patients were asked to report on their physical functioning and health related quality of life (13).

### 5.4. Recommendations

Since calcium overload significantly affects vascular calcification in dialysis patients, better survival may be achieved by maintaining a serum calcium level as low as possible within the standard range for patients on dialysis (14).

In our study, serum phosphorus level was found to be a strong contributor to hospitalization risk in adjusted model. Therefore, interventions designed to control serum phosphorus levels should be pursued more aggressively and encouraged in this population (29).

As a conclusion, in the current study, quality of life had correlation with serum calcium, calcium-phosphate product, and PTH concentrations, while hospitalization was correlated only with serum phosphorus level. However, quality of life had an inverse correlation with hospitalization period. Therefore, safe levels of serum calcium, phosphorus, and PTH need yet to be confirmed in further randomized control studies.
